# Albumin Is Synthesized in Epididymis and Aggregates in a High Molecular Mass Glycoprotein Complex Involved in Sperm-Egg Fertilization

**DOI:** 10.1371/journal.pone.0103566

**Published:** 2014-08-01

**Authors:** Kélen Fabíola Arroteia, Mainara Ferreira Barbieri, Gustavo Henrique Martins Ferreira Souza, Hiromitsu Tanaka, Marcos Nogueira Eberlin, Stephen Hyslop, Lúcia Elvira Alvares, Luís Antonio Violin Dias Pereira

**Affiliations:** 1 Department of Histology and Embryology, Institute of Biology, State University of Campinas (UNICAMP), Campinas, São Paulo, Brazil; 2 Department of Pharmacology, Faculty of Medical Sciences, State University of Campinas (UNICAMP), Campinas, São Paulo, Brazil; 3 ThoMSon Mass Spectrometry Laboratory, Department of Organic Chemistry, Institute of Chemistry, State University of Campinas (UNICAMP), Campinas, São Paulo, Brazil; 4 Faculty of Pharmaceutical Sciences, Nagasaki International University, Nagasaki, Japan; University of South Florida College of Medicine, United States of America

## Abstract

The epididymis has an important role in the maturation of sperm for fertilization, but little is known about the epididymal molecules involved in sperm modifications during this process. We have previously described the expression pattern for an antigen in epididymal epithelial cells that reacts with the monoclonal antibody (mAb) TRA 54. Immunohistochemical and immunoblotting analyses suggest that the epitope of the epididymal antigen probably involves a sugar moiety that is released into the epididymal lumen in an androgen-dependent manner and subsequently binds to luminal sperm. Using column chromatography, SDS-PAGE with *in* *situ* digestion and mass spectrometry, we have identified the protein recognized by mAb TRA 54 in mouse epididymal epithelial cells. The ∼65 kDa protein is part of a high molecular mass complex (∼260 kDa) that is also present in the sperm acrosomal vesicle and is completely released after the acrosomal reaction. The amino acid sequence of the protein corresponded to that of albumin. Immunoprecipitates with anti-albumin antibody contained the antigen recognized by mAb TRA 54, indicating that the epididymal molecule recognized by mAb TRA 54 is albumin. RT-PCR detected albumin mRNA in the epididymis and fertilization assays *in* *vitro* showed that the glycoprotein complex containing albumin was involved in the ability of sperm to recognize and penetrate the egg zona pellucida. Together, these results indicate that epididymal-derived albumin participates in the formation of a high molecular mass glycoprotein complex that has an important role in egg fertilization.

## Introduction

The epididymis is responsible for sperm concentration, transport and storage, but also promotes maturation by adding various proteins to the sperm surface [1]–[3]. Sperm maturation depends on the expression and secretion of proteins and glycoproteins by the epididymal epithelium, from the caput towards the cauda. These multiple and sequential interactions between the sperm surface and secreted proteins in the epididymal lumen are essential for the ability of mammalian sperm to fertilize oocytes [4]–[8].

The use of monoclonal antibodies (mAb) to study molecules expressed in male reproductive organs has contributed to our understanding of sperm maturation and the formation of a functional male gamete [9]–[12]. We have previously described the expression of a novel testicular (germ cell) [13] and epididymal [14] molecule using the mAb TRA 54 (**t**esticular **r**at **a**ntibody, obtained by immunizing rats with germ cells) [13]–[15]. The molecule identified by this antibody has a high molecular mass (∼260 kDa) and is resistant to treatment with SDS/β-mercaptoethanol [13], [14]. The epitope of the TRA 54 antigen probably contains carbohydrate domains as judged from its sensitivity to periodate [13], [14]. Immunohistochemistry revealed that the TRA 54-reactive molecule is located in the supranuclear cytoplasm of caput epididymal epithelial cells and also in luminal sperm. Overall, these results suggest that the molecule recognized by mAb TRA 54 is produced and released by epididymal epithelial cells and subsequently binds to the sperm surface as these cells move down the epididymal duct. The molecules recognized by mAb TRA 54 in epididymal epithelial cells are expressed independently of testicular germ cells and are produced in an androgen-dependent manner [14].

In this work, we used a combination of column chromatography, SDS-PAGE with *in* *situ* digestion and mass spectrometry to identify the protein recognized by mAb TRA 54 in mouse epididymal epithelial cells. Database searches and molecular modeling were used to identify the protein, and fertilization assays *in* *vitro* were used to examine the involvement of this protein in egg fertilization.

## Materials and Methods

### Animals

Male and female C57BL/6/JUnib mice (8 and 4 weeks old, respectively) were housed at 20–24°C on a 12 h light/dark cycle (lights on at 6∶00 a.m.) with free access to water and food. The mice were killed by cervical dislocation and their organs were processed immediately for subsequent experiments.

### Ethics statement

All of the animal experiments were approved by the institutional Committee for Ethics in Animal Experimentation (CEEA/UNICAMP, protocol no. 590-1) and followed the ethical guidelines of the Brazilian Society of Laboratory Animal Science (SBCAL).

### SDS-PAGE and immunoblotting

Caput epididymis was collected from adult C57BL/6 mice and homogenized in 1 ml of 10 mM Tris-HCl, pH 7.4, containing 10 mM EDTA (Mallinckrodt, Paris, France), 100 mM NaF, 10 mM Na_4_P_2_O_7_, 10 mM Na_3_PO_4_, 2 mM PMSF and 0.1 mg of aprotinin/ml (Sigma-Aldrich, St. Louis, MO, USA). The suspensions were subsequently centrifuged (10,000 *g*, 4°C, 15 min) and the resulting supernatant was centrifuged twice (25,000 *g,* 4°C, 25 min each). The protein concentration of the supernatants was determined using a Bradford protein assay kit (Bio-Rad, Richmond, CA, USA). Aliquots of each sample (100 µg per lane) were diluted in sample buffer and applied to 10% polyacrylamide gels followed by SDS-PAGE [16]. The proteins were subsequently transferred electrophoretically to polyvinylidenedifluoride (PVDF) membranes (Millipore, Bedford, MA, USA) for immunoblotting with mAb TRA 54 [13], [14] or pAb rabbit anti-albumin IgG (Abcam, Tokyo, Japan, ab34807) [17]. Immunoblotting was done to confirm the tissue distribution pattern of the immunoreactive bands before chromatography. The PVDF membranes were blocked with 5% low-fat dry milk for 1 h and then washed with Tris-buffered saline (150 mM NaCl in 50 mM Tris-HCl, pH 7.5) containing 0.05% Tween 20 (TBS-T). After incubation with mAb TRA 54 (1∶2,500 in TBS-T) or pAb rabbit anti-albumin IgG overnight at 4°C, the membranes were washed in TBS-T and incubated with goat anti-rat IgG or goat anti-rabbit IgG both conjugated with peroxidase (Dako A/S, Glostrup, Denmark) for 1 h at room temperature. After further washing, immunoreactive bands were visualized with an ECLplus staining kit (Amersham).

### Purification of the TRA 54-reactive protein

The mAb TRA 54-reactive antigen was purified from supernatants of epididymal homogenates by a combination of affinity chromatography and anion exchange chromatography. Initially, epididymal supernatant was loaded onto a Sepharose-concanavalin A (Con A) column (HR 5/20; Amersham Biosciences, Piscataway, NJ, USA) that specifically binds α-D-glucopyranosyl α-D-mannopyranosyl or sterically similar residues. The column was then washed with 0.1 M sodium acetate, pH 6.0, containing 1 M NaCl, 1 mM MgCl_2_ and 1 mM CaCl_2_. Bound proteins were eluted with a linear gradient of glucose (0–0.5 M) in the same buffer. The protein elution profile was monitored by measuring the absorbance at 280 nm (ÄKTApurifier10 chromatographic system, Amersham Biosciences) and fractions of 1 ml were collected automatically at flow rate of 1 ml/min.

The resulting fractions were screened for reactivity towards mAb TRA 54 by dot blotting and/or immunoblotting. For dot blotting, the fractions were spotted onto a previously activated nitrocellulose membrane (BioRad) and the membrane was processed for immunodetection using mAb TRA 54 [13], [14], essentially as described for immunoblotting. Immunoreactive fractions were pooled and concentrated by centrifugation (4,000 *g*, 4°C, 40 min) in Amicon 5 kDa membranes (Millipore) before anion exchange chromatography. The immunoreactive pool obtained in the previous step was applied to a HiTrap Q Sepharose High Performance column (Amersham Biosciences) previously equilibrated with 50 mM Tris-HCl, pH 7.4. Bound proteins were eluted with a linear gradient of NaCl (0–1 M) in this same buffer. Fractions that reacted with mAb TRA 54 (assessed by dot blotting) were combined to form three pools (A, B and C) based on their elution profile. The pools were concentrated by centrifugation (4,000 *g,* 4°C, 40 min) in Amicon 5 kDa membranes (Millipore) and analyzed by SDS-PAGE and immunoblotting with mAb TRA 54 to detect immunoreactive bands. Protein bands in the polyacrylamide gels were detected by staining with Coomassie blue.

### Gel in situ digestion with trypsin

The mAb TRA 54-reactive band detected in pool A was co-localized in the Coomassie blue-stained gel and excised after destaining. The excised fragment was diced and suspended in 0.2 M ammonium bicarbonate solution containing 45 mM dithiothreitol (DTT) for 30 min at 30°C. After centrifugation and removal of the supernatant the gel pieces were incubated with acetonitrile for 10 min and then dried under vacuum (Module 3180C speed vacuum dryer, Biotron, Puchon, Korea). The sample was rehydrated in 10 µl of 45 mM ammonium bicarbonate solution containing 0.5 µg of bovine trypsin (Sigma Aldrich) and incubated overnight at 37°C. The peptides generated by this digestion were extracted from the gel fragments by two incubations (at least 40 min each) with 100 µl of 0.1% trifluoroacetic acid (TFA) in 60% acetonitrile. Each incubation was followed by centrifugation to obtain a peptide-containing supernatant that was removed and dried by rotary evaporation [18].

### Protein identification by mass spectrometry (MS)

Mass spectra were acquired using matrix-assisted laser desorption ionization (MALDI) and time-of-flight (TOF) mass spectrometry (MALDI-TOF-MS) and a bidimensional capillary liquid chromatography (CapLC system, Waters, Manchester, UK) coupled to a tandem quadrupole *oa*-TOF hybrid mass spectrometer with a collision-induced dissociation (CID) hexapole cell (QTOF, Waters-Micromass, Manchester, UK) and a nano Z-spray source, both operated in positive ion mode. The TOF analyzer was operated in the reflectron mode.

### Peptide mass fingerprinting by MS

The samples obtained by tryptic digestion were prepared for analysis using the dried droplet method [18], [19]. Briefly, the samples were acidified by adding 1 µl of 0.1% (v/v) trifluoroacetic acid (TFA) in water followed by incubation for a few minutes at room temperature to allow reduction of the droplet volume by evaporation in the MALDI microplate. The matrix solution of 0.1% (w/v) α-cyano-4-hydroxycinnamic acid was prepared in a solution of 0.1% TFA with acetonitrile (1∶1, v/v) and 1 µl of this solution was added to the microplate. The sample was allowed to dry at room temperature. All of the measurements were done via MALDI-TOF-MS. Subsequent data evaluation and peptide identification were done using the Mascot Wizard software package (Matrix Science, London, UK; http://www.matrixscience.co.uk) [19].

### Reverse phase capillary liquid chromatography/electrospray ionization mass spectrometry (ESI-MS)

2D-LC-nano ESI-MS was done using a QTOF-1 instrument and a CapLC ternary pump system connected through a stream-select valve module to the nano Z-spray source. The sample was injected into the system through the CapLC autosampler using the “microliter pickup algorithm” injection mode. Solvent A consisted of 5% acetonitrile in 0.1% formic acid, solvent B consisted of 95% acetonitrile in 0.1% formic acid and solvent C consisted of 0.1% formic acid in H_2_O. The protein digest was pre-concentrated and desalted on a Waters Opti-Pak C_18_ trap column and connected to pump C through the stream-select valve module. The pre-concentration/desalting step was done at 5 µl/min over 20 min using pump C. After switching to pumps A and B, a gradient was applied to the precolumn cartridge and was then used to elute the sample from the capillary column (NanoEase; 75 µm i.d.×15 cm) packed with C_18_ resin (Waters). The column was equilibrated with 5% of solvent B and a linear gradient of this solvent (from 5% to 70%) was applied over 60 min (200 nl/min) using a pre-column split with the pump delivering at 15 µl/min. NanoESI-MS analysis over the *m/z* range of 300–2000 was done in-line with capillary chromatographic separation of the alkylated protein tryptic digests at a scan speed of 1 s/scan. Data-dependent acquisition (DDA) was done in-line with capillary chromatographic separation only for the alkylated protein tryptic digest. An initial nanoESI-MS survey was acquired over the *m/z* range of 300–2000 each second, with switching criteria for MS to MS/MS that included ion intensity and charge state. NanoESI-MS/MS data were acquired over the *m/z* range of 50–2000 each second for up to three co-eluting peptides but only for doubly and triply charged ions. Switch back from nanoESI-MS/MS to nanoESI-MS mode was allowed after 10 s. The collision energy used varied automatically according to the *m/z* ratio of the eluting peptides.

### Peptide sequencing by LC-nanoESI-MS/MS

The ionization conditions used included a capillary voltage of 2.8 kV and cone and RF1 lens voltages of 30 V and 100 V, respectively. The source temperature was 80°C and the cone gas was N_2_ at 0.15 bar. Argon was used to dissociate the ions in the hexapole collision cell. External calibration with [Glu]-Fib (Sigma-Aldrich F-3261) was done over the entire range of *m/z* 50–2000. All mass spectra were acquired with the TOF analyzer in reflectron “V-mode” (TOF kV = 7.2) and the MCP voltage was set at 2100 V.

### De novo sequencing of tryptic peptides

Alkylated tryptic peptides separated by reverse-phase capillary chromatography were lyophilized and resuspended in 20% acetonitrile in 0.1% formic acid prior to injection into the mass spectrometer source at a flow rate of 100 nl/min. The resulting product ion mass spectra were acquired using the TOF analyzer and deconvoluted using the MaxEnt3 algorithm. MS/MS data for singly charged ions was processed manually using the PepSeq application included in the MassLynx software package. The nanoESI-MS/MS data were processed using the softwares ProteinLynxGlobalServer v. 2.0.5 for peak-list (pkl)-generated output files and the Mascot MS/MS Ion Search module (http://www.matrixscience.co.uk).

### Computer-assisted alignment of the tryptic peptide sequences

The tryptic peptide sequences were aligned automatically by Mascot (http://www.matrixscience.co.uk) using the MOWSE scoring scheme. The peptide fingerprint analysis tool of Mascot incorporates a probability-based implementation of the MOWSE algorithm that accurately models the proteolytic behavior and specificity of trypsin. After scoring the data generated by the output files in Mascot, all of the peptides were used to search the databank for matching proteins followed by alignment with the protein that showed the best MOWSE-based probability scores [20].

### Amino acid sequence similarity search

The Basic Local Alignment Search Tool (BLAST) was used to compare the amino acid sequence obtained in the previous section with the sequences avaliable in the National Center for Biotechnology Information (NCBI) web site (http://www.ncbi.nlm.nih.gov).

### Immunoprecipitation assays

The pAb rabbit anti-albumin IgG (Abcam, Tokyo, Japan; ab34807) [17] or mAb TRA 54 (Abcam, Tokyo, Japan) [13], [14] was added to Dynabeads-protein G (Invitrogen, Tokyo, Japan), washed three times with washing buffer and cross-linked with dimethyl pimelimidate (DMP) buffer, followed by an additional three washes with 25 mM citrate/52 mM phosphate buffer (pH 5.0), according to the manufacturer’s instructions, prior to storage at 4°C. Caput epididymis was homogenized in lysis buffer (50 mM Tris-HCl, pH 7.5, 0.5 M sucrose, 1% dextran, 5 mM MgCl_2_) containing protease inhibitors – 1 mM EDTA and antipain, leupeptin and pepstatin A (3 µg/ml each). The lysates were centrifuged (200 *g*, 4°C, 10 min) and Dynabeads-protein G were incubated with 2.9 mg of supernatant protein for 40 min at 25°C followed by four washes with citrate-phosphate buffer (pH 5.0), according to the manufacturer’s instructions. Elution buffer (0.1 M sodium citrate, pH 3.1) was added to the Dynabeads-protein G and the samples then analyzed by western blotting. The protein concentration of each fraction was estimated based on the absorbance at 280 nm, with bovine serum albumin (BSA) as the standard. Subsequently, 50 µg of protein from each fraction was subjected to SDS-PAGE followed by electroblotting onto a polyvinylidenedifluoride (PVDF) membrane (Millipore). The membranes were blocked with 5% nonfat dry milk for 1 h at room temperature, washed for 15 min at room temperature with TBS (100 mM Tris-HCl, pH 7.5, 150 mM NaCl) containing 0.1% Tween-20, and then incubated with mAb TRA 54 [13], [14] or goat pAb against the N-terminal of albumin (Santa Cruz Biotechnology Inc., TX, USA; sc-46291) overnight at 4°C in Can Get Signal immunostain solution (Toyobo, Osaka, Japan). The membranes were then washed in PBS containing Tween 20 for 3 min followed by three 5 min washes prior to incubation with peroxidase-conjugated anti-rat immunoglobulins (1∶2.500; Invitrogen) for 1 h at 25°C. After further washing, immunoreactive bands were visualized with an ECLplus staining kit (Amersham).

### Reverse transcriptase-PCR (RT-PCR)

Total RNA was extracted from testis, total epididymis and epididymal regions (initial segment, caput, corpus and cauda) using Trizol reagent (Invitrogen, Maryland, USA) followed by treatment with DNAse I (Invitrogen, Maryland, USA) to eliminate contaminating genomic DNA. cDNA was synthesized using SuperScript III RNaseH - reverse transcriptase and oligo dT12-18 as primer. The albumin forward and reverse primers for PCR were: 5′-ATGAAGTGGGTAACCTTTCTCC-3′ and 5′-GGATGTCTTCTGGCAACTTC-3′, respectively. The reactions were run in a final volume of 25 µl containing 2 µl of cDNA, 10 pmol of primers, 0.2 mM dNTPs, 1.5 mM MgCl_2_, 1X PCR buffer, 2.5% DMSO and 1 U *Taq* DNA polymerase (LGC Biotechnology, London, United Kingdom). The PCR conditions consisted of an initial denaturation at 95°C for 1 min followed by five cycles of 95°C for 30 s, 55°C for 30 s and 72°C for 2 min, and 30 cycles of 95°C for 30 s, 55°C for 30 s and 72°C for 30 s. A final extension was done at 72°C for 10 min. One microliter of each PCR sample was electrophoresed in a 3% agarose gel, stained with ethidium bromide and photographed under ultraviolet light. The intactness and quality of total cDNA was checked by RT-PCR of the housekeeping gene cyclophilin. The identity of the albumin PCR product was confirmed by nucleotide sequencing (ABI 3730 DNA Analyser) and the size of the resulting fragment was determined by digestion using the restriction enzyme *Hinf*I (Fermentas Life Science, Burlington, ON, Canada). Fibrous cartilage (mouse tail) was used as a negative control and a liver extract as a positive control. The rections were done in triplicate.

### Immunolocalization and fate of the TRA 54 antigen during the acrosomal reaction

Fresh sperm were squeezed from mouse vasa deferentia into M16 medium (Sigma Aldrich) supplemented with 4 mg of BSA/ml. For capacitation, the spermatozoa were incubated for 2 h at 37°C in a 5% CO_2_ atmosphere and then pelleted by centrifugation (400 *g*, 10 min) in Dulbecco’s phosphate-buffered salt solution (Dulbecco’s PBS; Gibco). To induce the acrosomal reaction, capacitated sperm were resuspended in a solution containing the calcium ionophore A23187 (30 nM) for 20 min [21] and then washed three times in Dulbecco’s PBS by centrifugation (400 *g*, 10 min). For immunohistochemical analysis, 5 µl samples of the capacitated and acrosome-reacted sperm were dried on glass slides and fixed with ice-cold methanol for 30 min prior to immunostaining with mAb TRA 54 as previously described [13], [14], with slight modifications. Briefly, non-specific binding sites were blocked with 20% normal goat serum. The slides with capacitated or acrosome-reacted sperm were subsequently incubated overnight with mAb TRA 54 ascitic fluid (diluted 1∶4,000 in PBS/1% BSA) followed by incubation with a biotinylated anti-rat secondary antibody and detection with a Strept ABC kit (both from DAKO A/S, Glostrup, Denmark). The reaction was developed with hydrogen peroxide and diaminobenzidine. Control sections were treated similarly but without mAb TRA 54. To assess the status of the acrosome, slides containing capacitated or acrosome-reacted sperm were labeled with FITC-conjugated peanut agglutinin lectin (FITC-PNA) [22], [23]. Spermatozoa that showed no labeling were considered to have undergone a complete acrosomal reaction [24]. The resulting preparations were examined by fluorescence or phase-contrast microscopy using a Nikon microscope (Nikon, Tokyo, Japan).

### Sperm immunogold labeling with mAb TRA 54

Capacitated sperm or sperm that had undergone an acrosomal reaction were washed three times in PBS by gentle centrifugation and fixed in 1 ml of 4% paraformaldehyde and 0.2% glutaraldehyde in 0.1 M sodium cacodylate buffer, pH 7.4, at 4°C for 1 h. The samples were then washed three times in 0.05 M Tris-HCl, pH 7.4, and dehydrated in a graded series of N-N-dimethylformamide and embedded in LR-White resin at −20°C for 48 h to ensure complete polymerization. Ultrathin sections (100 nm) were collected on nickel grids, washed for 5 min in 0.05 M Tris-HCl, pH 7.4, and preincubated for 30 min with 0.05 M Tris-HCl, pH 7.4, containing 1% BSA. The grids were subsequently incubated overnight at 4°C with mAb TRA 54 (diluted 1∶3,000) followed by washing with 0.05 M Tris-HCl, pH 7.4, and incubation for 1 h with a goat anti-rat secondary antibody conjugated to gold particles (15 nm diameter) (Dako A/S). The sections were stained with uranyl acetate. Gold-labeling control reactions were done by omitting the incubation with mAb TRA 54.

### Effect of the TRA 54-reactive protein on the acrosomal reaction

Sperm from vasa deferentia were distributed in four groups (I, II, III and IV) and incubated in M16-BSA for 2 h at 37°C in a 5% CO_2_ atmosphere (12 determinations per group). Sperm in group I were incubated only in M16-BSA, group II was incubated with 1% mAb TRA 54 [13], [14], group III was incubated with 1 µM calcium ionophore A23187 and group IV was treated with both mAb TRA 54 and calcium ionophore (the antibody was added before the ionophore). The sperm acrosomal status was assessed by FITC-PNA [22], [23]. The percentage of sperm with intact acrosomes or that underwent the acrosomal reaction in each group was determined by fluorescence microscopy (excitation at 494 nm; emission at 520 nm). Fluorescence-negative sperm were considered to be completely acrosome-reacted [24]. For statistical analysis, at least 400 sperm were counted in each condition or treatment.

### *In vitro* fertilization assays

Female mice (4 weeks old) were superovulated by consecutive intraperitoneal injections of 5 IU of equine chorionic gonadotropin (eCG; Syntex S.A., São Paulo, SP, Brazil) and 48 h later with 5 IU of human chorionic gonadotropin (hCG; Calier, Barcelona, Spain). The stimulated females were mated with vasectomized males after hCG administration. Ovulated oocytes were collected by rupturing oviductal ampullae 20 h after hCG administration. The oocytes were immediately transferred to M2 culture medium pre-equilibrated at 37°C in a 5% CO_2_ atmosphere. The cumulus complexes were removed from oocytes by brief treatment with 0.01% hyaluronidase in M2 [25]. Some cumulus-free oocytes were briefly incubated with low pH (2.5) Tyrode solution [26] to remove the zona pellucida (zona pellucida-free oocytes). The zona pellucida-intact and zona pellucida-free oocytes were transferred to drops of M16-BSA medium in 35 mm Falcon dishes under mineral oil. For fertilization, capacitated or acrosome-reacted spermatozoa obtained by treatment with a nanomolar concentration of the calcium ionophore A23187 [27] were distributed in three samples: control group I - incubated for 3 h in M16-BSA medium alone, control group II - incubated in medium containing 10% rabbit normal serum, and test group - incubated in medium containing 10% mAb TRA 54. The final spermatozoa concentration was adjusted to 2×10^6^ spermatozoa/ml in a 60 µl drop of medium. The zona pellucida-intact oocytes were co-incubated with capacitated spermatozoa and the zona pellucida-free oocytes were co-incubated with acrosome-reacted spermatozoa. After 5 h of co-incubation at 37°C in a 5% CO_2_ atmosphere the oocytes and adhered spermatozoa were transferred to a fresh drop of medium. Fertilization was assessed using an inverted microscope based on the presence of two cells after 24 h of gamete co-incubation. Each assay was done 6–12 times.

### Statistical analysis

The numerical data for sperm acrosomal status were expressed as the mean ± SEM and were analyzed statistically by one-way analysis of variance (ANOVA) followed by the Tukey test, with p<0.05 indicating significance. The results for the IVF assays were analyzed using the chi-square (χ^2^) test, with a value of p<0.01 indicating significance after application of the Bonferroni correction for multiple comparisons.

## Results

### Detection of a TRA 54-immunoreactive high molecular mass complex in total caput epididymis

**Figure 1:**
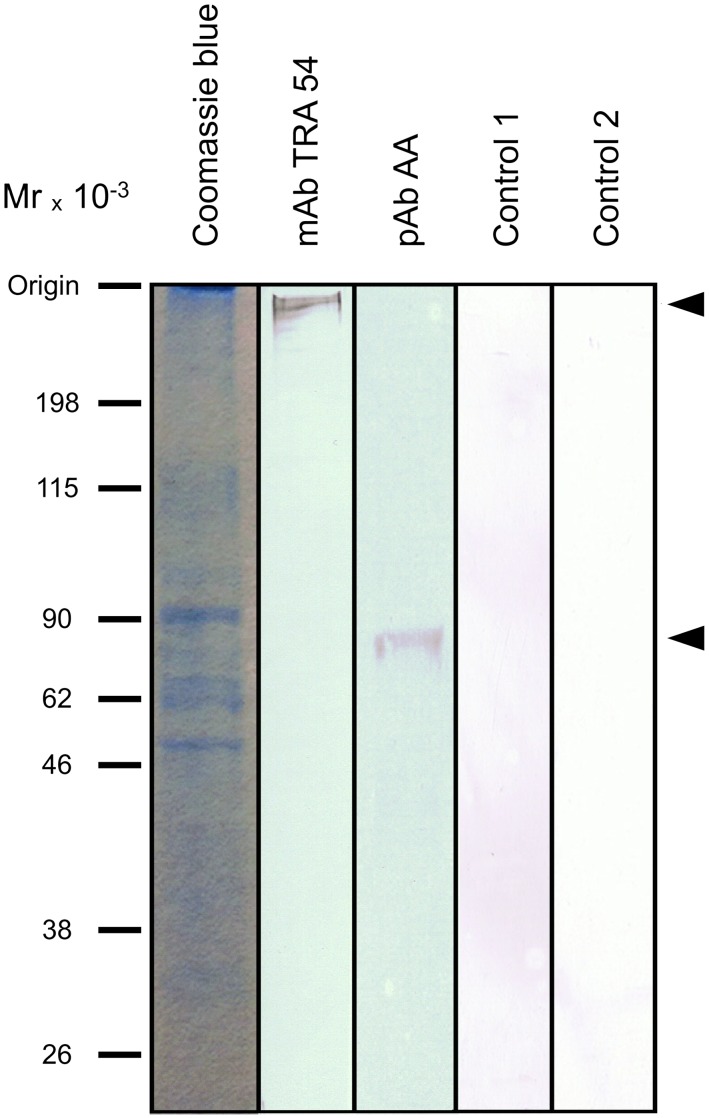
Immunoblots of mouse caput epididymis extracts. Aliquots (50 µg) of protein were electrophoresed in a 10% polyacrylamide gel and transferred to a PVDF membrane for immunoblotting with mAb TRA 54 or pAb rabbit anti-albumin (Abcam, Tokyo, Japan, ab34807). Arrows indicate the upper (mAb TRA 54) and lower (pAb anti-albumin) limits of the protein complexes detected in the samples. Control 1 is an immunoblot without mAb TRA 54 and control 2 is a kidney extract immunoblotted with mAb TRA 54. No immunoreactive bands were detected in either of these controls. Molecular mass markers (MM) are shown on the left.

Western blots of C57 BL/6 caput epididymis extracts revealed a band of ∼260 kDa that reacted with mAb TRA 54 (Fig. 1).

### Purification of the epididymal protein recognized by mAb TRA 54

**Figure 2:**
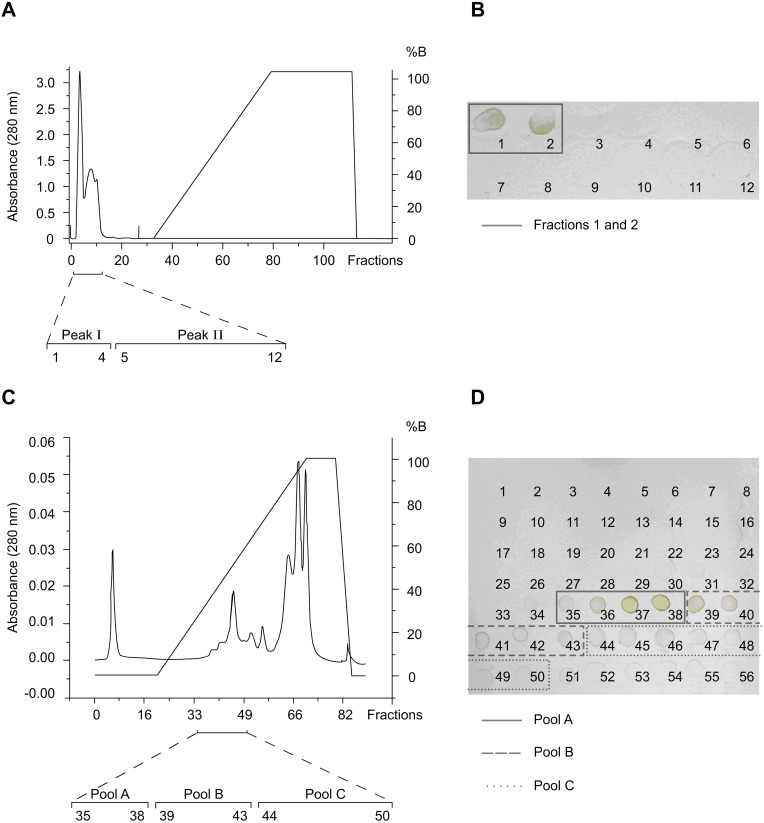
Partial purification of the mouse epididymal protein detected with mAb TRA 54. A) Fractionation of a homogenate of epididymal caput tissue on a column of Sepharose-concanavalin A (Con-A). After washing the column with 0.1 M sodium acetate, pH 6.0, containing 1 M NaCl, 1 mM MgCl_2_ and 1 mM CaCl_2_, the column was eluted with a linear gradient (0–0.5 M) of glucose in this same buffer. Note that two peaks (I and II) eluted during column washing and no proteins were eluted with the glucose gradient. B) A dot blot of the fractions corresponding to peaks I and II showed that only fractions 1 and 2 of peak I reacted with the mAb TRA 54. These fractions were combined and purified further by anion exchange chromatography (HiTrap Q-Sepharose high performance column). C) Elution profile of immunoreactive fractions 1 and 2 (from peak I of the Sepharose-Con A step) after ion exchange chromatography. Bound proteins were eluted with a linear gradient of NaCl (0–1 M) in 50 mM Tris-HCl, pH 7.4. D) A dot blot identified several fractions that reacted with mAb TRA 54. The positive fractions were combined to form three pools (pool A: fractions 35–38; pool B: fractions 39–43; pool C: fractions 44–50) that were subsequently analyzed by electrophoresis and immunoblotting.

**Figure 3:**
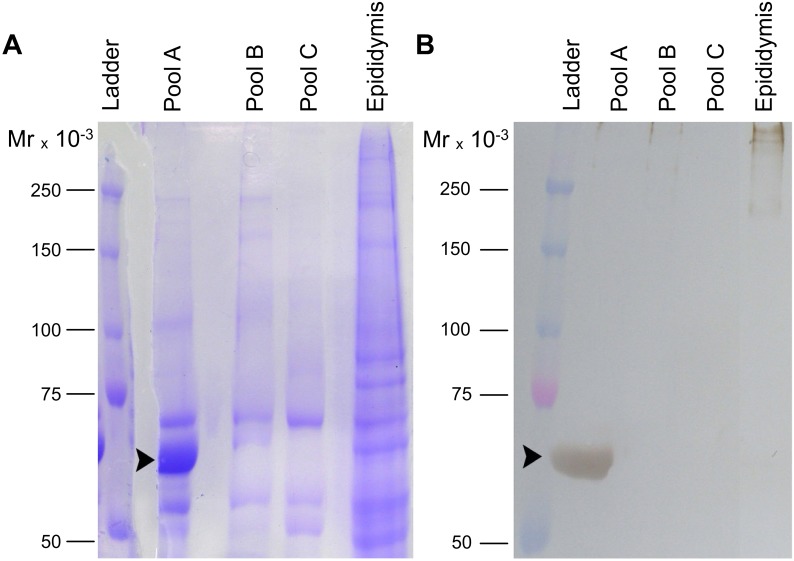
SDS-PAGE and immunoblot of the purified mouse epididymal protein. Aliquots (100 µg) of immunoreactive pools A, B and C obtained by ion exchange chromatography and epididymal extracts were electrophoresed in a 7.5% polyacrylamide gel (A) and transferred to a PVDF membrane for immunoblotting with mAb TRA 54 (B). A strongly immunoreactive band of ∼65 kDa was detected in pool A (arrowhead in panel B), but not in pools B and C. The corresponding protein band in the polyacrylamide gel stained with Commassie blue (arrowhead in panel A) was excised and the protein digested with trypsin. Control immunoblots without mAb TRA 54 showed no bands (not shown). MM – molecular mass markers.

Affinity (Con A) chromatography of caput epididymis homogenates yielded two peaks of unbound material, of which only fractions (tubes) 1 and 2 of the first peak reacted with mAb TRA 54; no additional proteins were eluted with the glucose gradient, indicating that under these conditions there was little protein interaction with the column (Fig. 2A, B). Subsequent anion exchange chromatography of the positive fractions (Fig. 2C) followed by dot blot analysis identified three immunoreactive groups: fractions 35–38, 39–43 and 44–50 that were combined to form pools A, B and C, respectively (Fig. 2C, D). SDS-PAGE (Fig. 3A) and immunoblotting (Fig. 3B) of pool A showed a ∼65 kDa band that reacted strongly with mAb TRA 54; this band was not detected in pools B and C. The protein band corresponding to the immunoreactive band in pool A was excised from the Commassie blue-stained gel for subsequent digestion with trypsin.

### Identification of the TRA 54-reactive epididymal protein as albumin based on peptide sequencing by mass spectroscopy (MS) and immunoprecipitation

Tryptic peptides of the purified protein band were detected by MALDI-TOF-MS and sequenced by LC-nanoESI-MS/MS. Incubation of the purified protein with trypsin yielded 17 peptides for peptide mass fingerprinting and 15 for *de* *novo* sequencing from MS/MS spectra. Subsequent searches of the NCBI databases using the tryptic peptide sequences yielded strong matches with mouse albumin-1, a major circulating protein in blood. Figure S1 compares the sequence of this protein with mouse albumin-1. The protein sequence has been deposited in the UniProt Knowledgebase under accession number P07724.

**Figure 4:**
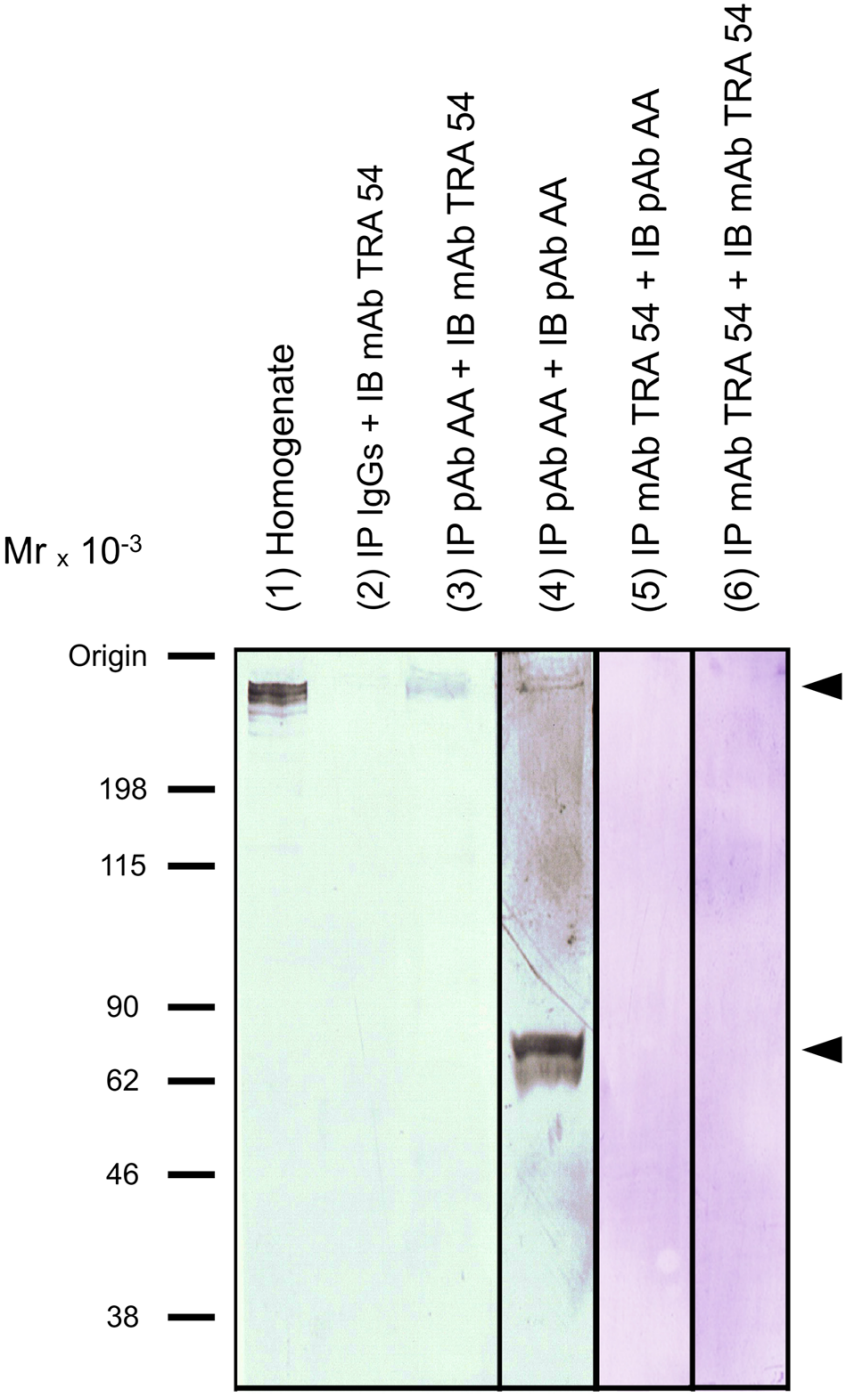
Identification of the mAb TRA 54 and pAb anti-albumin-reactive protein in mouse caput epididymis by immunoprecipitation followed by immunoblotting. Detection of TRA 54-reactive protein in caput epididymis homogenates. Aliquots (50 µg) of the homogenate was loaded (lane 1). The immunoprecipitate (IP) obtained with IgGs was probed (IB) with mAb TRA 54 (lane 2). The immunoprecipitate obtained with pAb rabbit anti-albumin (AA) IgG (Abcam; ab34807) was probed with mAb TRA 54 (lane 3) or with goat pAb against the N-terminal of albumin (Santa Cruz Biotechnology; sc-46291) (lane 4). The immunoprecipitate obtained with mAb TRA 54 was probed with rabbit anti-albumin IgG (Abcam; ab34807) (lane 5) and mAb TRA 54 (lane 6). For immunoprecipitation, Dynabeads-protein G (Veritas, Tokyo, Japan) were used according to the manufacturer’s recommendation. Arrows indicate the signals. Molecular mass markers are indicated on the left.

To confirm that albumin was the antigen that reacted with mAb TRA 54, immunoblotting with mAb TRA 54 was done after immunoprecipitation of proteins from caput epididymis with anti-albumin pAb IgG antibody or mAb TRA 54 cross-linked to Dynabeads-protein G. The immunoprecipitates obtained from caput epididymis obtained with anti-albumin pAb antibody and probed with mAb TRA 54 resulted in the detection of a TRA 54-reactive protein (∼260 kDa), further confirming that the epididymal molecule recognized by mAb TRA 54 contained albumin (Fig. 4, lane 3). The immunoprecipitates obtained from caput epididymis using anti-albumin pAb and probed with a goat pAb against the N-terminal of albumin included the detection of a TRA 54-reactive protein (∼260 kDa) and an ∼65 kDa fraction, further confirming the indentification of albumin as part of a high molecular mass complex (Fig. 4, lane 4). In addition, a faint immunoreactive band was observed at ∼260 kDa when immunoprecipitation and immunoblotting were done with mAb TRA 54 (Fig. 4, lane 6).

### mRNA for albumin is synthesized by epididymal and testicular cells

**Figure 5:**
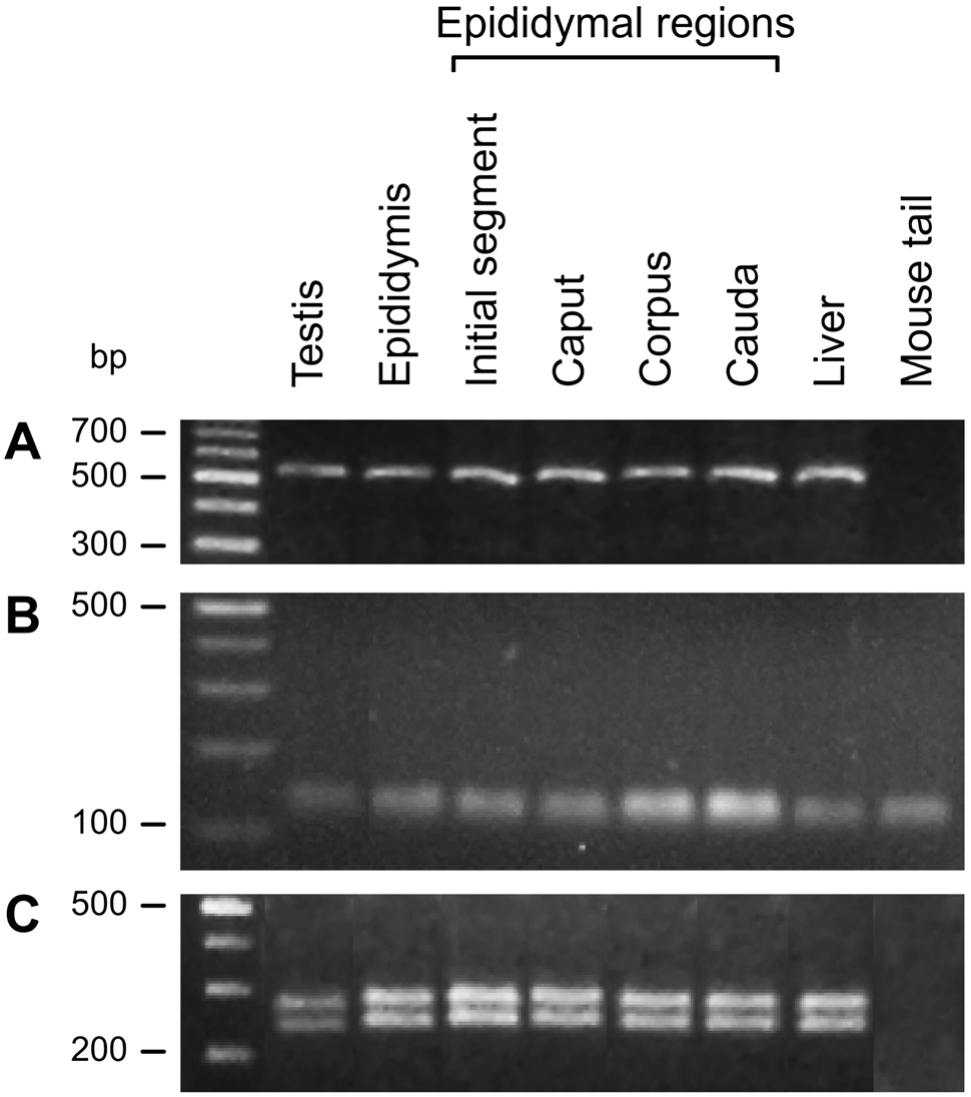
Albumin mRNA transcripts detected by RT-PCR. (A) RT-PCR detected albumin mRNA in mouse testis and epididymis (and all regions of the latter). In these assays, mouse liver was used as a positive control and fibrous cartilage from mouse tail was used as a negative control. The PCR products were analyzed by electrophoresis in 3% agarose gels and included a 100 bp DNA ladder. (B) The housekeeping gene was used to check the intactness and quality of total cDNA. The forward and reverse primers for PCR were: 5′-CTTGCTGCAGACATGGTC-3′ and 5′-GCAATCCTGCTAGACTTG-3′, respectively. The blank was used as a control RT-PCR reaction. The PCR products were analyzed by electrophoresis in 3% agarose gels and included a 100 bp DNA ladder. (C) The size of the albumin PCR fragment was confirmed by digestion using the restriction enzyme HinfI. The digestion fragments were analyzed by electrophoresis in 3% agarose gels and included a 100 bp DNA ladder.

RT-PCR showed that albumin mRNA was transcribed in testis and epididymis, including all regions of the latter (Fig. 5A). The expression of the housekeeping gene cyclophilin confirmed the intactness of the cDNA (Fig. 5B). The nucleotide sequences (BankIt1558664, 1562898 and 1562932) and the restriction enzyme digestion profile confirmed the PCR product to be albumin (Fig. 5C).

### The high molecular mass glycoprotein complex containing albumin is present in the sperm acrosome

**Figure 6:**
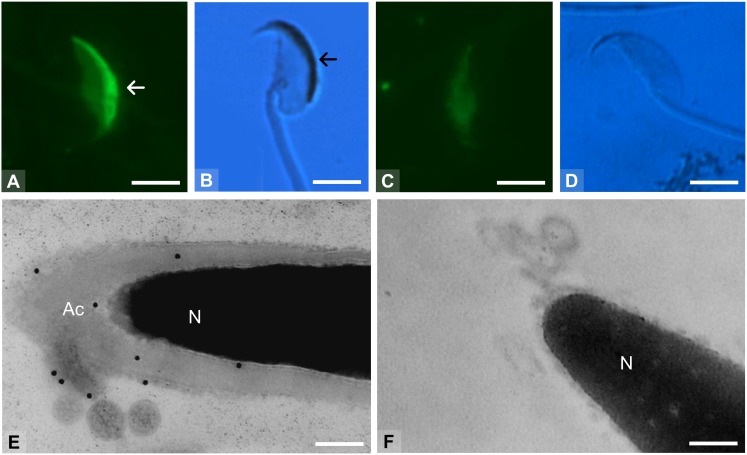
Expression of the TRA 54 antigen in sperm based on immunoreactivity with mAb TRA 54. The integrity of the sperm acrosome was evaluated by the presence (arrow in A) or absence (C) of a FITC-PNA fluorescent area in the sperm head. The TRA 54 antigen was detected within the acrosome in capacitated and intact sperm (arrow in B), but was not seen after the acrosomal-reaction (D). The sperm flagellum was not labeled (B). (E, F) Transmission electron microscopy (TEM) of sperm immunolabeled with mAb TRA 54. (E) Positive reaction (gold particles) in the plasma membrane that covered the sperm caput and in the acrosome (Ac) of capacitated sperm. (F) No TRA 54 antigen was detected in acrosome-reacted sperm. N – nucleus. Panels (A) and (C) are fluorescent images, (B) and (D) are phase-contrast images, and (E) and (F) are TEM images. Note that the images in panels (A) and (B) and in panels (C) and (D) are from the same preparations but with different illumination. Scale bars: A–D –3.5 µm, E–0.2 µm, F–0.35 µm.

Intact sperm labeled with FITC-PNA showed no acrosomal reaction (Fig. 6A). The acrosomal region but not the flagellum or other regions of the sperm contained the TRA 54 antigen (Fig. 6B). Sperm without fluorescent staining were considered to have undergone a complete acrosomal reaction (Fig. 6C) and no positive reaction to mAb TRA 54 was seen in these cells (Fig. 6D). Transmission electron microscopy of immunogold-labeled preparations showed a positive reaction to mAb TRA 54 in the plasma membrane surrounding the sperm head and in the acrosome (Fig. 6E). Acrosome-reacted sperm showed no positive labeling with mAb TRA 54 (Fig. 6F).

### The high molecular mass glycoprotein complex containing albumin is involved in sperm penetration of the zona pellucida

**Figure 7:**
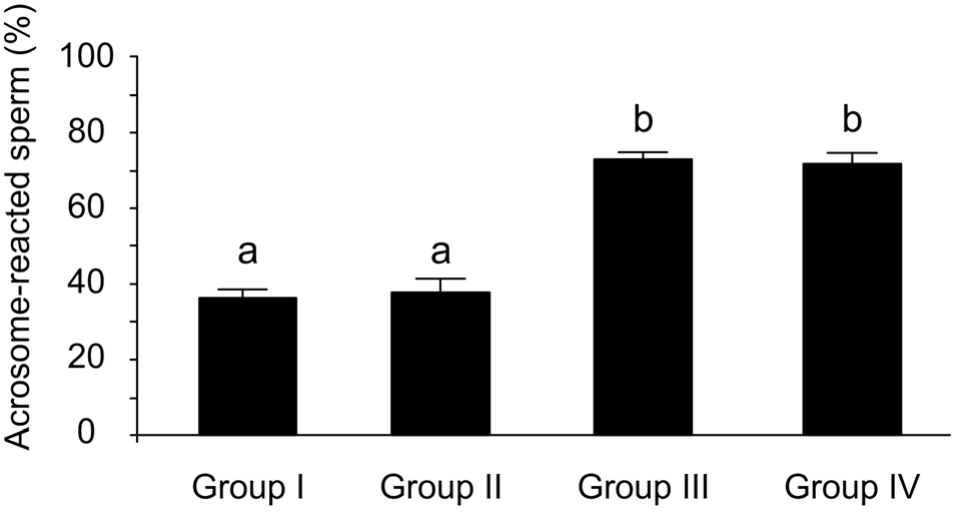
Effect of mAb TRA 54 on the acrosomal reaction induced by the calcium ionophore A23187. Group I – control sperm, group II – mAb TRA 54 added to sperm, group III – calcium ionophore added to sperm and group IV – mAb TRA 54 added to sperm before calcium ionophore. The columns are the mean ± SEM (n = 12 determinations/sample). Means with different letters differed significantly (F = 119, 3; p<0.05).

Prior addition of mAb TRA 54 to sperm did not alter the acrosomal reaction rate to calcium ionophore A23187 (Fig. 7). When zona pellucida-intact oocytes were used for *in vitro* fertilization assays, the fertilization rate was 83.9% (n = 47 out of 56) in the control group and 83.7% (n = 41/49) in the group receiving rabbit normal serum (control groups I and II, respectively; χ^2^ = 0.001; p = 0.97), but only 30.2% (n = 16/53) when mAb TRA 54 (test group) was added (χ^2^ = 32.24, p<0.0001 for test group versus control group I; χ^2^ = 29.54, p<0.0005 for test group versus control group II).

Sperm in the three groups (control group I, control group II and test group) were equally able to fertilize zona pellucida-free oocytes since the fertilization rates were 86.9% (n = 53/61), 81.3% (n = 26/32) and 83.1% (n = 49/59), respectively (χ^2^ = 0.52, p = 0.47 for control group I versus control group II; χ^2^ = 0.35, p = 0.56 for test group versus control group I and χ^2^ = 0.05, p = 0.83 for test group versus control group II).

## Discussion

Several molecules secreted into epididymal fluid contribute to the surface modifications involved in sperm maturation [1]–[5]. We have previously described an epididymal molecule recognized by mAb TRA 54 that appeared to be released from epididymal epithelial cells and subsequently adhered to luminal sperm [14]. The peculiar expression pattern of this protein suggested that, among other possible functions [13], this molecule could be involved in sperm-egg recognition or egg penetration. In the present study, we sought to identify the epididymal protein involved in the reactivity with mAb TRA 54 and investigate its possible involvement in fertilization.

By using a combination of affinity (lectin) and anion exchange chromatography, SDS-PAGE with *in* *situ* digestion, mass spectrometry and immunodetection (dot blotting and western blotting) we identified a ∼65 kDa protein that reacted with mAb TRA 54. Curiously, the major mAb TRA 54 immunoreactive band of caput epididymis homogenates had a molecular mass of ∼260 kDa, which is considerably different from the ∼65 kDa found for the isolated epididymal protein. This difference in size suggest that the isolated molecule recognized by mAb TRA 54 is part of an SDS/β-mecaptoethanol-resistant complex of higher molecular mass that may contain more than one type of protein or may be modified by the addition of oligosaccharide chains. We suggest that the ∼260 kDa protein complex underwent dissociation during purification, as observed for other protein complexes [28]. The presence of glycosidic components in the molecular complex recognized by mAb TRA 54 [13]–[15] could contribute to the formation of molecular aggregates typically found in the epididymal lumen [4], [29]–[32] and in the sperm acrosome [24].

Mass spectrometric analysis of the epididymal protein recognized by mAb TRA 54 and its tryptic fragments by MALDI-TOF-MS and 2D-LC-nanoESI-MS/MS, respectively, yielded a molecular mass of 64,961 Da, which agreed with the ∼65 kDa initially estimated by SDS-PAGE. Comparison of the peptide sequences obtained here with those in the NCBI database revealed a match between the epididymal protein recognized by mAb TRA 54 and mouse albumin-1.

Immunoprecipitates of caput epididymis homogenates using anti-albumin pAb IgG included the molecule recognized by mAb TRA 54. This finding confirmed that the high molecular mass complex identified by mAb TRA 54 included albumin and probably other protein or glycosidic components.

The presence of albuminoid proteins in the epididymis has been described by others [29], [33], who have identified prealbumin epididymal-specific (PES) proteins as a class of epididymal proteins characterized by the presence of structural domains related to the albumin domains [28], [34]. PES isoforms have been identified in ovine [28], ram [34], [35] and rat [33], [36], [37] tissue. Dacheux *et al.* [38] detected albumin in human epididymis, but this protein was considered a serum component rather than a molecule synthesized by the epididymal epithelium. To clarify whether the testis and epididymis are actually sites of albumin biosynthesis (as opposed to being a serum complex-component transported to these organs) we assessed the gene expression of albumin in both organs by RT-PCR. Our results confirmed that albumin mRNA is synthesized by testicular cells [39] but also provided the first evidence for albumin gene expression in epididymal cells, i.e., this gene is expressed in these two organs in adult mice. Despite evidence suggesting that albumin is expressed in epididymal tissue [33], [34], no previous work has shown that albumin is indeed synthesized by epididymal cells. The four regions of the epididymis expressed the albumin gene, indicating that the entire epididymis and, consequently, all stages of sperm maturation, may be dependent on or modulated by a high molecular mass glycoprotein complex containing albumin. This finding is particularly interesting since, in addition to the classic sites of albumin expression (such as liver and yolk sac [40], [41]), new sites of mRNA transcription for albumin have been described in recent years, including the mammary gland, tongue, intestine, lymph node, testis, and uterus of bovines [39], [42], as well as the brain [43] in humans.

In the male reproductive tract, albumin may be involved in the transport of other molecules to the sperm membrane during epididymal maturation, in the movement of proteins during the acrosomal reaction, or in membrane remodeling during sperm-oocyte membrane fusion [36]. Epididymal albumin may also be responsible for assembling the (glyco) protein complex recognized by mAb TRA 54. In addition, the epididymal albumin-containing protein complex may be absorbed by the sperm acrosome, as occurs with other epididymal secreted proteins [24]. Although the high molecular mass complex is not required to trigger the acrosomal reaction, this complex is gradually dispersed and fully exocytosed during the reaction. This forward movement of proteins could involve albumin as a carrier molecule.

In rats, PES proteins are released from the epididymis in a testosterone-dependent manner, can bind to the sperm membrane when these cells pass through the epididymal lumen [14], [28], [29], [33], [44] and may participate in sperm binding to the zona pellucida [36]. We hypothesize that the same pattern of synthesis and function might apply to the complex recognized by mAb TRA 54 [13], [14] and that, in addition to epididymal PES, epididymal albumin may have an important role in fertilization after exocytosis of the albumin-containing complex during the acrosomal reaction.

In agreement with this suggestion, the *in* *vitro* fertilization experiments described here showed that the addition of mAb TRA 54 to the fertilization medium significantly inhibited the fertilization rate of zona pellucida-intact oocytes but did not affect this rate in zona pellucida-free oocytes. Together, these findings indicate that the epididymal high molecular mass complex containing albumin is involved in the optimization of zona pellucida penetration by an acrosome-reacting sperm. In addition, epididymal albumin could facilitate the transportation and coupling of other molecules and enzymes that become tightly bound to the sperm surface and are required for the secondary binding of sperm to the oocyte membrane [24], [44], [45].

In conclusion, we have identified a high molecular mass complex containing albumin in homogenates of caput epididymis. In addition, albumin mRNA was detected in testes and epididymis. Functional assays involving fertilization *in* *vitro* demonstrated that this high molecular mass complex has a role in fertilization. Since infertility in mice and humans is strongly associated with a lack of epididymal molecules [46]–[48], and since the high molecular mass complex containing albumin characterized here is also expressed by human epididymis (unpublished observations), identification of the other molecules present in the epididymal complex recognized by mAb TRA 54 could be helpful in understanding male infertility.

## Supporting Information

Figure S1 **Amino acid sequence of the protein and its alignment with human and mouse albumin-1 proteins.** The amino acid sequence of the protein was identified by LC-nanoESI-MS/MS.
(TIF)
