# Supplementary figures and images for: Albumin Is Synthesized in Epididymis and Aggregates in a High Molecular Mass Glycoprotein Complex Involved in Sperm-Egg Fertilization
Source: PLoS One. 2014 Aug 1;9(8):e103566. doi: 10.1371/journal.pone.0103566 (PMC4118885; doi:10.1371/journal.pone.0103566)

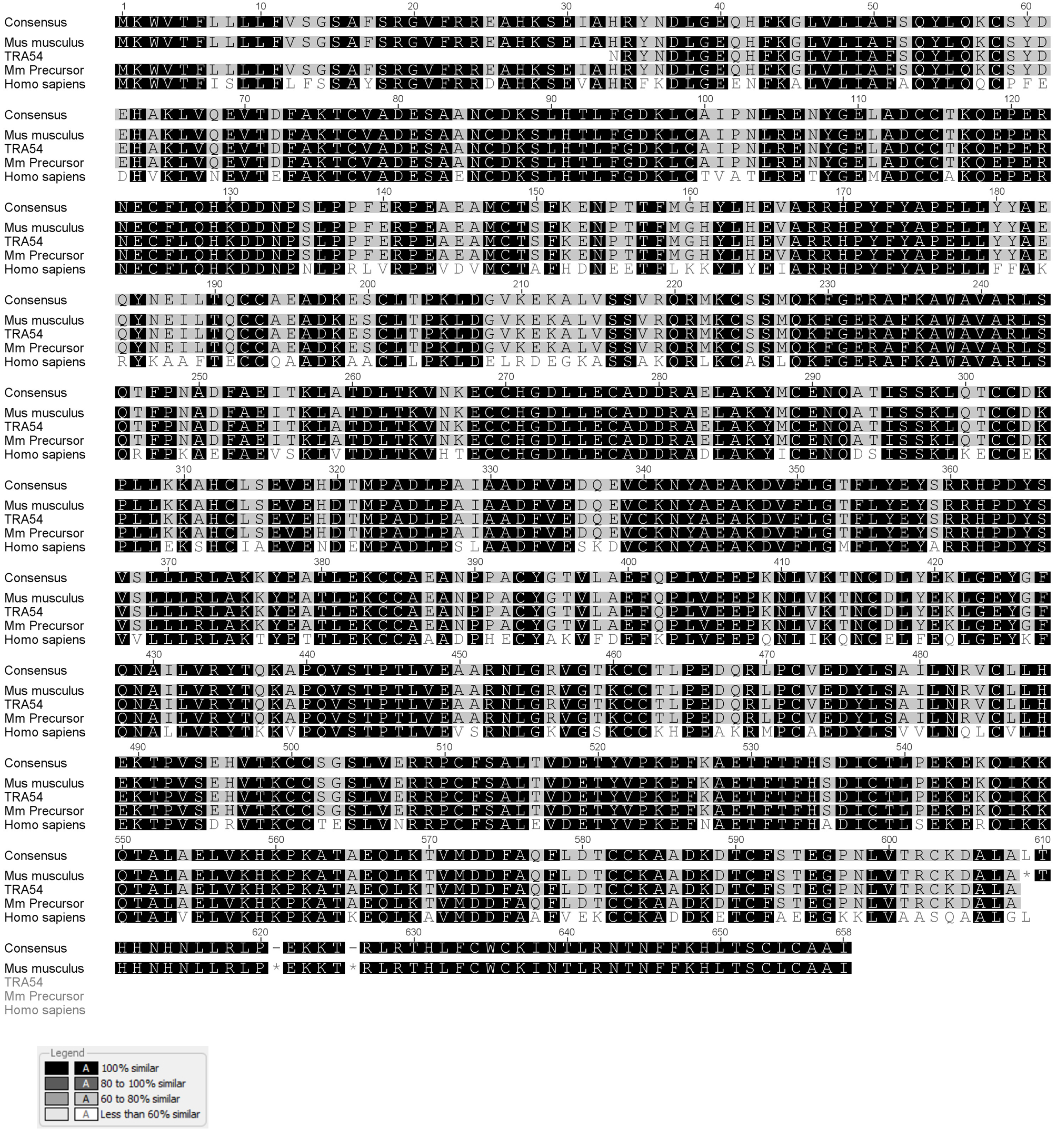

Supplement: Figure S1 — Amino acid sequence of the protein and its alignment with human and mouse albumin-1 proteins. The amino acid sequence of the protein was identified by LC-nanoESI-MS/MS. (TIF) [file pone.0103566.s001.tif]
